# Prediction of Emergency Department Re-Visits in Older Patients by the Identification of Senior at Risk (ISAR) Screening

**DOI:** 10.3390/geriatrics3030033

**Published:** 2018-06-21

**Authors:** Ksenija Slankamenac, Gertraud Haberkorn, Otto Meyer, Heike A. Bischoff-Ferrari, Dagmar I. Keller

**Affiliations:** 1 Emergency Department, University Hospital Zurich, Raemistrasse 100, 8091 Zurich, Switzerland; gertraud.haberkorn@usz.ch (G.H.); dagmar.keller@usz.ch (D.I.K.); 2 Department of Geriatrics, University Hospital Zurich, University Zurich, Centre on Aging and Mobility, Raemistrasse 100, 8091 Zurich, Switzerland; otto.meyer@ksb.ch (O.M.); heike.bischoff@usz.ch (H.A.B.-F.)

**Keywords:** re-visit, geriatric assessment, emergency medicine, Identification of Seniors at Risk (ISAR) screening

## Abstract

The “Identification of Seniors at Risk” (ISAR) screening is a tool to identify seniors at risk of adverse outcomes. We investigated whether seniors with a positive ISAR screening have an increased risk of Emergency Department (ED) re-visits and health-service costs. In a pilot project, we enrolled 96 ED patients (≥70 years) who received an ISAR screening in the ED. We compared the rate of ED re-visits and in-hospital costs between ISAR positive (≥2 pts) and ISAR negative (<2 pts) patients. In some patients, a geriatrician performed a single Geriatric Consultation (GC) during the ED stay to assess older patients’ needs.32% of the study population had an unplanned ED re-visit (31 of 96). Fifty patients were ISAR positive (52%) and showed an increased risk of ED re-visits compared with ISAR negative patients (dds ratio (OR) 6.8, 95% confidence interval (CI) 2.2–21.0, *p* = 0.001). The positive ISAR screening tool fairly predicted ED re-visits in seniors (area under the curve (AUC) 0.711). A single GC during the ED stay did not reduce the risk of unplanned ED re-visits in ISAR positive patients (*p* = 0.80) ISAR positive patients with GC did not have higher in-hospital costs than ISAR negative patients without GC. Based on these findings, we aim to establish a comprehensive outpatient geriatric assessment program to identify relevant risk factors for ED re-visits and to recommend preventive strategies in ISAR positive ED seniors.

## 1. Introduction

The ageing of the population and the increased prevalence of chronic-degenerative diseases, falls, as well as exacerbations of co-morbidities make older people frequent users of Emergency Departments (ED) [1,2]. Older patients often suffer from atypical clinical presentations, and in combination with multiple co-morbidities and cognitive limitations, the ED management and treatment of these patients becomes highly complex [3,4]. Thus, older patients are at increased risk of adverse outcomes, such as unplanned ED re-visits, hospital readmission, and even death [3]. Therefore, different attempts were made to decrease the risk of ED re-visits in seniors. For example, one method is the two-stage nursing assessment and intervention to early identify seniors’ problems after ED discharge [5]. Despite a wide range of research projects, unplanned ED re-visits in seniors stayed a major issue.

There are simple and valid tools to predict adverse health outcome in the ED and to identify older patients at risk [6]. The “Identification of Seniors at Risk” (ISAR) screening predicts a wide range of adverse health outcomes, including death, hospital readmission, resource use, and physical or cognitive function [7]. The ISAR score includes six simple dichotomous questions, making it short, simple, and acceptable for patients and staff alike [6]. If patients have two or more positive answers (≥2 points) in the ISAR screening, further geriatric assessment and treatment is recommended to improve the prognosis and to reduce the risk of adverse outcome [8]. Even though the ISAR screening is an established and widely used screening method, it is also discussed controversially in the literature. Its predictive validity related to mortality and adverse outcomes was graded as poor to fair [7,9,10]. Nevertheless, the ISAR screening tool for ED seniors at risk definitely has a clinical value, even if the prognostic performance is not completely satisfactory [8,11,12,13,14,15].

The so-called Comprehensive Geriatric Assessment (CGA) includes a multidisciplinary approach for diagnostic and treatment methods to define medical, psychosocial, and functional limitations, and to manage resources for the frail seniors with the goal of improving seniors’ health outcome [16]. CGA of the overall health status begins with a screening process using simple, inexpensive, and internationally validated scales to rate cognitive function, functional status, walking, balance, and socio-economic status [16]. The CGA presents a significant support after hospital discharge and is associated with a reduction in hospital readmission, mortality, and replacement to nursing homes of older people [16,17,18]. Nevertheless, CGAs in the ED are time-consuming and beyond the scope of most EDs; they are therefore performed during the hospital stay or in an outpatient setting. Therefore, we started a pilot project with symptom- and cause-related Geriatric Consultations (GCs) during the ED stay in June 2015. In this pilot project, we aimed to offer the ED seniors a modern, comprehensive, and interdisciplinary assessment and ED therapy. Based on this pilot project, the aim of this study was to investigate whether an increased ISAR score predicts adverse outcome, and whether a single GC during an ED stay may reduce both the rate of ED re-visits within one month after the index ED treatment and health service costs in older ED patients in a tertiary Swiss hospital.

## 2. Methods

We retrospectively investigated the data from a pilot project enrolling irregularly older patients (≥70 years) who were admitted in the ED of a tertiary care hospital from June 2015 to January 2016. Although many studies on the ISAR screening were performed in patients aged 65 or older [8,11,12,14,19], there are also studies showing that the ISAR prediction is better in older patients with a cut-off age of 70 and older [7,20]. Furthermore, there are studies performing the ISAR screening on patients aged 75 and older [21]. Due to these opposed data on the cut-off of age in the ISAR screening, we and the geriatricians involved decided by mutual agreement to use the ISAR screening in ED patients aged 70 and older.

We only enrolled patients when a geriatrician was personally present in the ED. In general, the geriatrician was present once to three times per week from Monday to Friday, 8 a.m. to 5 p.m. The geriatrician performed all assessments of seniors and did the single GCs. In some situations, although a geriatrician was present in the ED, not every ISAR positive ED patient could be assessed by the geriatrician. The reasons for this were when the geriatrician was already occupied in another geriatric assessment, an urgent medical assessment could not be delayed or the health condition of the patient did not allow a geriatric assessment.

We completed the ISAR screening tool [8] in all of these ED seniors during their ED stay. Patients who had two or more positive answers were reported as ISAR positive (≥2 pts), whereas patients with none or only one positive answer were ISAR negative (<2 pts) [22,23].

The study was approved by the local ethic committee (2016-00715).

### 2.1. Endpoints

We investigated as the primary endpoint the incidence of unplanned ED re-visits within one month after the index ED visit. We defined ED re-visits as consultations within 30 days after index ED discharge or after hospital discharge. In all of the patients in our study, ED re-visits after hospital discharge happened to be within 30 days after index ED visit. Furthermore, we evaluated the predictive validity of the ISAR screening for ED re-visits. As secondary endpoints, we investigated the 30-day mortality rate, the rate of placements to nursing home, and the need for hospital stay. Moreover, we investigated the in-hospital costs of those older patients who need to be admitted to the hospital.

### 2.2. Assessment of Other Study Variables

Additionally, to the primary and secondary endpoints, we retrospectively ascertained clinical and demographic data, as well as functional parameters from the electronic clinical information system and electronic medical records in order to characterize the included patient population. We assessed the following parameters: triage at admission by the Emergency Severity Index (ESI) [24], age, sex, body mass index, primary diagnosis, co-morbidities, Charlson co-morbidity index, signs of malnutrition, (instrumental) activities of daily living (independent and dependent), depressive disorders, cognitive impairments, alcohol syndrome, poly-pharmacy (<6 drugs per day and ≥6 drugs per day), and social data, such as residency (home alone, home with others, nursing home, or retirement home), family status (single, married, or widow/-er).

### 2.3. The Symptom- and Cause-Related Geriatric Consultations (GC)

A Comprehensive Geriatric Assessment (CGA) in the ED is time-consuming and beyond the scope of the ED. Therefore, a geriatrician from the Test- and Diagnostic Center of the Department of Geriatrics of the Zurich University Hospital performed a symptom- and cause-related Geriatric Consultation (GC) during the ED stay. The geriatrician focused on needs and deficiencies that were obviously associated with the underlying disease and reason for the ED presentation. In every patient beyond the underlying disease, the geriatrician checked and optimized the medication concerning poly-pharmacy, gave recommendations to prevent falling, immobility and malnutrition, and registered those patients for further out- or inpatient medical assessments, individual physiotherapy, or even rehabilitation. In general, the single GC took 30–60 min to complete. If there was time in the ED, the geriatrician individually extended the examinations based on the co-morbidities by using the general instruments of CGA such as the mini mental state examination, confusion assessment method, geriatric depression scale, or Katz index for Activities of Daily Living (ADL).

### 2.4. Statistical Analysis

In a first step, we expressed the distribution of variables using means and Standard Deviation (SD) for normally distributed data, and medians and Interquartile Ranges (IQR) for non-normally distributed data. Categorical data were presented as frequencies.

We presented the primary endpoint (prevalence of unplanned ED re-visits within one-month after index ED visit) and all secondary endpoints as proportions. We compared all endpoints between patients with negative ISAR screening (<2; control group) and positive ISAR screening (≥2, experimental group) using univariate and multivariable logistic regression models, adjusting for potential confounders such as sex, emergency severity index, Charlson co-morbidity index, and performed GC in the ED. No adjustment and therefore no multivariable regression analysis was performed if less than five cases occurred in both groups. Confounding factors were chosen a priori based on clinical interest and scientific knowledge.

We also focused on patients receiving a single GC during their ED stay, and compared the outcome (unplanned ED re-visits within one month after index ED visit) between both groups (ISAR negative and positive patients) with the help of univariate logistic regression analysis. Due to the small sample size in this subgroup analysis, a controlling for potential confounders was not allowed.

Furthermore, we compared the first endpoint (unplanned ED re-visits within one-month after index ED visit) between patients receiving a single GC during the ED stay and the ones who did not receive a GC using univariate and multivariable logistic regression analysis adjusting for sex, emergency severity index, Charlson co-morbidity index, and positive ISAR screening.

The diagnostic value of the ISAR tool (predictive validity) for the adverse outcome “re-visit” was analyzed using the Receiver-Operating-Characteristic (ROC) curve. The Area Under the Curve (AUC) was interpreted as such [25]: excellent: 0.90–1.00; good: 0.80–0.89; fair: 0.70–0.79; poor: 0.60–0.69; and no value: 0.50–0.59. For all results, we reported point estimates, 95% confidence intervals, and *p*-values (<0.05 considered significant). We performed the statistical analyses using the statistical program STATA SE (version 15, Stata Corp., College Station, TX, USA).

## 3. Results

During the pilot project, we performed the ISAR screening on 96 ED patients, of which 50 were screened ISAR positive (≥2 pts.) and 46 ISAR negative (<2 pts.). The mean age of patients was 83 years (SD 6.7 years.). Fifty-three patients were male (55.2%). Twenty-seven patients (28.1%) were in the ED because of a trauma, whereas 71.9% came with symptoms or aggravation of an internal medical disease. The waiting time until treatment in the ED was similar in both groups (Table 1). The mean treatment time in the ED of all patients was 313 min (SD 146 min), of which ISAR positive patients had a longer treatment time than ISAR negative patients (338 min (SD 125 min) vs. 285 min (SD 164 min)) (Table 1).

Focusing on the descriptive characteristics between both groups (negative vs. positive ISAR), the ESI was similar between the groups (Table 1). ISAR positive patients had more internal medical problems (80%) than ISAR negative patients (63%). In 52% of the ISAR positive patients, the Charlson Co-Morbidity index was higher compared with ISAR negative patients (13%). Signs of malnutrition, poly-pharmacy and cognitive disorders were more often present in ISAR positive patients (Table 1). ISAR positive patients also needed more often support and were less independent in the activities of daily living than ISAR negative patients (Table 1). More than a quarter of ISAR positive patients (26%) lived in a nursing or retirement home (Table 1).

Almost a third of our study population (32.3%) had an unplanned ED re-visit within 30 days after the index ED visit (Table 2). ISAR positive patients had a significantly increased risk of unplanned ED re-visits within 30 days compared with ISAR negative patients (50% vs. 13%, adjusted OR 6.5, 95% CI 2.1–20.1, *p* = 0.001). Other outcome parameters, such as 30-day mortality, need for hospital stay, and need for nursing home care after discharge were similar in both groups (Table 2). Analyzing the predictive validity, ISAR positive screening predicted fairly unplanned ED re-visits at one month after the index ED presentation (AUC 0.711, 95% CI 0.607–0.797).

In 29 patients (30.2%), a geriatrician performed a first symptom- and cause-related GC during the ED stay. Eighteen (62.1%) of these 29 patients with a GC were ISAR positive, of which six experienced an unplanned ED re-visit. Following the single GC during the ED stay, ISAR positive seniors showed a higher rate (44.4% vs. 18.2%) of unplanned ED re-visits than ISAR negative patients; the difference was not significant (unadjusted OR 3.6, 95% CI 0.6–21.6, *p* = 0.16) (Table 3). Furthermore, senior patients receiving a single GC during the ED stay did not show a significantly reduced risk of unplanned ED re-visits (OR 0.9, 95%-CI 0.3–2.6, *p* = 0.80) (Table 4) compared with senior patients who did not have a GC during the ED stay.

Furthermore, the in-hospital costs were neither increased nor decreased in those patients receiving a single GC (9202 CHF (IQR 3958–16,457 CHF)) compared with those who did not receive (11,212 CHF (IQR 4837–18,140 CHF) the GC (adjusted difference −4803 CHF, 95% CI −19,196–9590 CHF, *p* = 0.50).

Focusing on the results of these 29 GCs, we can report that in 82.7% of these patients (*n* = 24) the geriatrician initiated a drug therapy reduction due to drug interactions or even stopped it because there was no need. Seventeen patients (58.6%) received a prescription for care home support and help. In 13 patients (44.8%), an early complex therapy was recommended. Eleven patients received recommendations and further prevention of fall and supportive materials. The geriatrician prescribed an evidence-based home training in one third of these patients (34.4%), whereas nine other patients (31%) needed an acute inpatient geriatric hospital treatment. Eight patients (27.6%) were planned for further outpatient testing: four patients (13.8%) for osteoporosis, and another four for cognitive impairment. In six patients, a recommendation for a better nutrition was given, whereas six other patients (20.7%) needed a change in residency and were planned for a placement in a nursing home.

## 4. Discussion

Senior ED patients have multiple and complex health problems and need more time and more resources than younger ED patients [3,4]. Seniors experienced more adverse outcomes following an ED treatment and had therefore an increased risk of ED re-visits, hospital admission, or even mortality [3]. In our pilot study, we could show that almost a third of our seniors had an unplanned ED re-visit within 30 days after index ED treatment. The ED re-visits in seniors were fairly predictable with the positive ISAR screening tool. A single GC during the ED stay did not reduce the risk of unplanned ED re-visits in ISAR positive patients. Regardless of GCs during the ED stay, in-hospital costs were not increased in those patients receiving a single GC.

In the literature, there is a wide range in the rate of unplanned ED re-visits due to different definitions of the endpoint. Some authors investigated unplanned ED re-visits within 72 h, seven days, 30 days, or even three to six months following the index ED presentation [3,7,8,11,14,26,27]. Compared with the one-month period, the rate varies from 10 to 25% [3,8,11,14,26,27,28]. In our pilot study, we had a 32% rate of unplanned ED re-visits. One reason for the increased rate of unplanned ED re-visits might be that we manage as many patients as possible in an outpatient setting. Switzerland has an excellent outpatient follow-up care system provided by general physicians, making it possible to discharge the seniors. In some cases (e.g., during weekends or nights), this treatment strategy failed and resulting in ED re-visits. Another reason is that all eight seniors who died within 30 days were managed in an outpatient setting. All of them had unplanned ED re-visits within 30 days. It is known that end-stage patients have unplanned and repeated ED re-visits in the month before dying [29]. These two causes increased our rate of unplanned ED-revisits.

There are few studies investigating the predictive validity of the ISAR screening for unplanned ED re-visits after one month following the index ED visit [8,11,14]. In 2000, McCusker et al. reported a 19.3% rate of ED re-visits in the first 30-day after index ED presentation and showed that one item of the ISAR screening tool (hospitalization in the past six months) significantly predicted the 30-day ED re-visits, while the predictive validity of the model was poor (AUC 0.63) [8]. In 2009, Salvi et al. presented that the ISAR screening tool did not predict the 30-day ED re-visits [11]. Three years later, the same research group showed a prediction of the ED re-visits by the ISAR screening tool, but with a poor validity (AUC 0.63) [14]. Other published studies predicted the risk of ED re-visits after 4 or 6 months and could only show a fair to poor validity for the ISAR screening tool [11,12,13,14]. Predicting the ED re-visits after 4–6 months in an aging populating is too long from our point of view, because the risk of unplanned ED re-visits after 4–6 months is not directly associated with the first symptoms, usually due to co-morbidities and the natural process of diseases. Therefore, we investigated the ISAR screening as a one-month predictor for unplanned ED re-visits in our ED seniors and could present a fair predictive validity of the ISAR screening tool for early ED re-visits. In contrast, there are studies predicting the risk of hospital readmission in older ED patients after one month [8,19,26]. Those studies showed consistent predictive validity of the ISAR screening for the prediction of unplanned ED re-visits, just as our study did. Furthermore, an Italian research group focused on the six-month outcome and could show that ISAR positive patients have an increased 6-month risk of death, long-term care placement, functional decline, ED re-visits, and hospital admission [11]. The same research group showed in 2012, that an ISAR screening may be used to select high-risk patients more likely to benefit from a geriatric approach or intervention, independently of admission or discharge [14].

In a randomized controlled trial in 2004, Caplan et al. showed that a CGA reduced the rate of ED re-visits within an 18-month follow-up period and prolonged the time to the first ED admission after the index ED treatment [18]. Ellis et al. recently published in 2014 that CGA in the ED improved the adverse outcomes by reducing the rate of hospital admission, mortality, and dependence [30]. A systematic review by Graf et al. confirmed that CGA in ED is efficient in decreasing ED readmission, functional decline, and possibly nursing home admission in high-risk patients [31]. However, the same group also showed that a routinely performed CGA in the ED is too time-consuming and therefore may not be performed consecutively [31]. In contrast, there were also studies that did not show any effect of GCs during the ED stay on the rate of ED re-visits [5,32]. These results are similar to our analysis of GCs on the rate of ED re-visits. We could not show any effect of single GCs on the reduction of unplanned ED re-visits in general when comparing patients with and without a GC during the ED stay. In a subgroup analysis, we could show that a single GC during the ED stay reduced the risk of unplanned ED re-visits from 6.5 to 3.6, comparing ISAR positive and negative screened patients. The rate of unplanned ED re-visits in ISAR positive patients was also reduced by almost 5%. GCs seem to have an impact only on a specific and selected patient population with ISAR positive screening. This analysis was based on a low number of ED patients and might have a selection bias. This needs further investigations and randomized controlled trials to properly answer this question.

It is known that a CGA during a hospital stay has a positive impact in patients’ outcome, such as reducing the length of hospital stay and increasing the functional status or patient satisfaction [33,34]. Many studies also showed that CGAs are cost effective [34,35,36]. However, several studies also reported cost differences due to differences in length of hospital stay, need for intensive care, or differences in the type as well as number of investigations requested between patients receiving a CGA compared with the control group [37]. Cohen at al. demonstrated that if the nursing home costs were taken into consideration, the benefit of a performed CGA was much higher [34]. Although we studied a limited number of patients, we were able to show that in-hospital costs were not increased in those patients receiving a single GC compared with those who did not receive a GC during the ED stay.

Interestingly, we found an increased treatment time of ISAR positive seniors (338 min (SD 125 min) vs. 285 min (SD 164 min)). This increase in time is due to the time-consuming nature of the GC and not due to more diagnostics during the ED stay.

Our study has several limitations. First, it is a pilot project in a single tertiary care center on a low number of older ED patients. Because such a GC is time-consuming and need a patient who is in a moderate enough condition to answer the questions, do our tests, and pass examinations, we only recruited 96 patients in the pilot project. Furthermore, the low sample number is also caused by the limited presence of the geriatrician in the ED, which may constrain the generalizability of our results. Nevertheless, the results of this study are in the line with the literature. A further limitation is that we only considered patients who re-visited our ED, but we missed all other geriatric patients who came to other EDs, hospitals, or had unplanned visits to the general physician.

Nonetheless, we successfully investigated the risk of unplanned ED re-visits after one month following the index ED treatment. In an aging population, a narrower endpoint such as one month is necessary to investigate the unbiased impact on the ED re-visits. The longer the observing time is, the more likely new diseases or exacerbations of co-morbidities are biasing the primary results. A second strength of our study is that a multivariable regression analysis was performed to control for possible confounders, which reduced the bias in this study.

## 5. Conclusions

Older ISAR positive patients had an increased risk of unplanned ED re-visits. A single GC did not reduce unplanned ED re-visits in seniors. Regardless of GCs in the ED, a single GC did not increase in-hospital costs in those patients receiving a single GC during the ED stay. Based on these findings, further investigations are needed and we therefore aim to establish a comprehensive outpatient geriatric assessment program to identify relevant risk factors for ED re-visits and to recommend preventive strategies in ISAR positive ED seniors.

## Figures and Tables

**Table 1:** Patients’ characteristics.

Characteristics	All Patients *n* = 96	ISAR Negative (<2 pts.) *n* = 46	ISAR Positive (≥2 pts.) *n* = 50
Age (years)	82.8 (6.7)	82.4 (6.9)	83.2 (6.5)
Sex, male/female (%)	53/43	25/21	28/22
	55.2%/44.8%	54.3%/45.7%	56%/44%
Reason for ED visit *:			
- injury	27 (28.1%)	17 (37.0%)	10 (20%)
- cardio-vascular	21 (21.9%)	8 (17.4%)	13 (26%)
- infection	16 (16.7%)	8 (17.4%)	8 (16%)
- neurology	12 (12.5%)	7 (15.2%)	5 (10%)
- abdominal incl. urogenital and kidney	12 (12.5%)	8 (17.4%)	4 (8%)
- pulmonary	10 (10.4%)	3 (6.5%)	7 (14%)
- bleeding	4 (4.2%)	1 (2.2%)	3 (6%)
- malignancy	3 (3.1%)	1 (2.2%)	2 (4%)
- other	1 (1.0%)	1 (2.2%)	0%
Emergency Severity Index (ESI)			
- 1	0%	0%	0%
- 2	11 (11.5%)	3 (6.5%)	8 (16%)
- 3	79 (82.3%)	38 (82.6%)	41 (82%)
- 4	6 (6.3%)	5 (10.9%)	1 (2%)
- 5	0%	0%	0%
Waiting time in the ED (minutes)	15 (16)	17 (15)	13 (17)
Treatment time in the ED (minutes)	313 (146)	285 (164)	338 (125)
Charlson co-morbidity index (≥4)	32 (33.3%)	6 (13.0%)	26 (52%)
Signs of malnutrition (%)	15 (15.6%)	5 (10.9%)	10 (20%)
Polypharmacy	6.1 (4.5)	3.5 (2.8)	8.6 (4.4)
- ≥6 drugs daily	49 (51.0%)	10 (21.7%)	39 (78%)
Cognitive impairment (%)	19 (19.8%)	3 (6.5%)	16 (32%)
- new diagnosed	4 (4.2%)	1 (2.2%)	3 (6%)
Acute delirium (%)	7 (7.3%)	3 (6.5%)	4 (8%)
Depressive disorder (%)	9 (9.4%)	4 (8.7%)	5 (10%)
Alcohol syndrome (%)	9 (9.4%)	5 (10.9%)	4 (8%)
Activities of Daily Living (ADL)			
- Independent	69 (71.9%)	41 (89.1%)	28 (56%)
- Need some support/assistance	20 (20.8%)	4 (8.7%)	16 (32%)
- dependent	7 (7.3%)	1 (2.2%)	6 (12%)
Instrumental Activities of Daily Living (iADL)			
- Independent	66 (69.8%)	40 (87.0%)	26 (52%)
- Need some support/assistance	22 (22.9%)	4 (8.7%)	18 (36%)
- dependent	8 (8.3%)	2 (4.3%)	6 (12%)
Residence (%)			
- home alone	29 (30.2%)	19 (41.3%)	10 (20%)
- home with others	54 (56.3%)	27 (58.7%)	27 (54%)
- nursing home	8 (8.3%)	0%	8 (16%)
- retirement home	5 (5.2%)	0%	5 (10%)
Marital status			
- married/partner	56 (58.3%)	25 (54.3%)	31 (62%)
- divorced/separated	7 (7.3%)	4 (8.7%)	3 (6%)
- widowed	28 (29.2%)	15 (32.6%)	13 (26%)
- single	5 (5.2%)	2 (4.3%)	3 (6%)

All results were reported in mean (standard deviation); ISAR = Identification of Seniors at Risk; ED = Emergency Department; * some patients had more than one diagnosis.

**Table 2:** Outcome Analysis if ISAR (<2/≥2).

Outcome	ISAR Negative (<2 pts.) *n* = 46	ISAR Positive (≥ 2 pts.) *n* = 50	Unadjusted OR (95% CI, *p*-Value)	Adjusted OR (95% CI, *p*-Value)
Unplanned ED re-visits (%)	6 (13.0%)	25 (50%)	6.7 (2.4–18.5, *p* < 0.001)	6.8 (2.2–21.0, *p* = 0.001)
Mortality within 30 days (%)	1 (2.2%)	7 (14%)	7.3 (0.9–62.1, *p* = 0.068)	-
Need for hospital stay (%)	24 (52.2%)	39 (78%)	3.3 (1.3–7.9, *p* = 0.009)	1.8 (0.6–5.3, *p* = 0.273)
Need for nursing home care (%)	2 (4.3%)	4 (8%)	1.9 (0.3–10.9, *p* = 0.467)	-

ISAR = Identification of Seniors at Risk; OR = Odds Ratio; CI = Confidence Interval; ED = Emergency Department. All results were adjusted for sex, Charlson co-morbidity index (<4/≥4), emergency severity index and performed geriatric consultation in the ED (no/yes). No adjustment was performed if less than five cases occurred in each group.

**Table 3:** Outcome analysis in patients if a single geriatric consultation was performed during the ED stay.

Outcome	ISAR Negative (<2 pts.) *n* = 11	ISAR Positive (≥2 pts.) *n* = 18	Unadjusted OR (95% CI, *p*-Value)	Adjusted OR (95% CI, *p*-Value)
Unplanned ED re-visits (%)	2 (18.2%)	6 (44.4%)	3.6 (0.6–21.6, *p* = 0.16)	-

ISAR = Identification of Seniors at Risk; OR = Odds Ratio; CI = confidence Interval; ED = Emergency Department; No adjustment was performed if less than five cases occur in each group.

**Table 4:** Outcome analysis concerning the effect of a single geriatric consultation during the ED stay.

Outcome	No GC During ED Stay *n* = 67	GC during ED Stay *n* = 29	Unadjusted OR (95% CI, *p*-Value)	Adjusted OR (95% CI, *p*-Value)
Unplanned ED re-visits (%)	21 (31.3%)	10 (34.5%)	1.2 (0.5–2.9, *p* = 0.76)	0.9 (0.3–2.6, *p* = 0.80)

GC = Geriatric Consultation; OR = Odds Ratio; CI = Confidence Interval; ED = Emergency Department; All results were adjusted for a sex, Charlson co-morbidity index (<4/≥4), emergency severity index and positive Identification of Seniors at Risk screening.

## Abbreviations

ADL	Activities of Daily Living
AUC	Area Under the Curve
CGA	Comprehensive Geriatric Assessment
CHF	Swiss Francs
CI	Confidence Interval
ED	Emergency Department
ESI	Emergency Severity Index
GC	Geriatric Consultation
ISAR	Identification of Seniors at Risk
IQR	Interquartile Range
pts	points
ROC	Receiver-Operating-Characteristic
OR	Odds Ratio
SD	Standard Deviation
